# Expression of Inflammatory and Cell Death Program Genes and Comet DNA Damage Assay Induced by *Escherichia coli* in Layer Hens

**DOI:** 10.1371/journal.pone.0158314

**Published:** 2016-06-27

**Authors:** Gamal M. K. Mehaisen, Mariam G. Eshak, M. I. El Sabry, Ahmed O. Abass

**Affiliations:** 1 Department of Animal Production, Faculty of Agriculture, Cairo University, Giza, Egypt; 2 Department of Cell Biology, National Research Centre, Giza, Egypt; Wageningen UR Livestock Research, NETHERLANDS

## Abstract

Modern methods of industrial poultry and egg production systems involve stressful practices that stimulate *Escherichia coli* (*E*. *coli*) activity causing endotoxic shock. This investigation was conducted to evaluate the expression of pro-inflammatory cytokines and cell death program genes and DNA damage induced by *E*. *coli* in the brain and liver tissues of laying hens. A total of two hundred and ten H&N brown layer hens with 20 week age, were used in this research. First, preliminary experiments were designed (60 hens in total) to establish the optimal exposure dose of *E*. *coli* and to determine the nearest time of notable response to be used in the remainder studies of this research. At 35-wk of age, 150 hens were randomly assigned into 2 groups with 3 replicates of 25 birds each; the first group was injected in the brachial wing vein with 10^7^ *E*. *coli* colony/hen, while the second group was injected with saline and served as a control. The body temperature and plasma corticosterone concentration were measured 3 hr after injection. Specimens of liver and brain were obtained from each group and the gene expression of *p38* mitogen-activated protein kinase, interlukin-1β (*IL-1β*), tumor necrosis factor alpha (*TNF-α*), *Bax*, and *caspase-3* genes were measured by quantitative real-time PCR. DNA damage in the brain and liver tissues were also measured by comet assay. Hens treated with *E*. *coli* showed significant (P<0.05) increase of body temperature and plasma corticosterone (42.6°C and 14.5 ng/ml, respectively) compared to the control group (41.1°C and 5.5 ng/ml, respectively). Additional remarkable over-inflammation gene expression of *p38*, *IL-1β* and *TNF-α*.genes were also detected in the brain (2.2-fold, 2.0-fold and 3.3-fold, respectively) and the liver (2.1-fold, 1.9-fold and 3.0-fold, respectively) tissues of the infected chickens. It is also important to note that hens injected with *E*. *coli* showed an increase in DNA damage in the brain and liver cells (P<0.05). These results were synchronized with activating cell death program since our data showed significant high expression of *Bax* gene by 2.8- and 2.7-fold and *caspase-3* gene by 2.5- and 2.7-fold in the brain and liver tissues of infected chickens, respectively (P<0.05). In conclusion, the current study indicates that *E*. *coli* injection induces inflammatory physiological response and triggers cell death program in the brain and liver. Our results provide more understanding to endotoxic shock by *E*. *coli* in chickens at cellular level. Further studies are required to confirm if such responses are destructive or protective to set the means through which a chicken mounts a successful defense against avian pathogenic *E*. *coli*.

## Introduction

It is estimated that in order to feed the world population in 2050, there has to be a 70% increase in food production over today’s levels. The livestock sector, particularly poultry production, forms an essential component to meet these demands in both developed and undeveloped regions. Despite the improvement in poultry production systems and the parallel enhancement in the trade volume of their products over the past years, Avian Pathogenic *Escherichia Coli* (APEC) is the leading cause of morbidity and mortality in poultry and exerts significant economic and welfare costs and continues to pose a formidable challenge to poultry industry. For example, the commercial egg laying industry in US is comprised of over 303 million laying hens in April 2016, of which about 263 million table eggs are produced per day [1]. Because of massive egg production, virtually laying hens are exposed to a wide range of potential stressors including cages, housing-specific challenges, disease agents, poor bone strength, balanced rations, foot health, and pests and parasite load [2]. Such stress leads to activate *E*. *coli* in the digestive system of chickens which in turn induces endotoxin stress [3]. Clinically, APEC is a strain of *E*. *coli* that has the ability to enter the host through ingestion or inhalation, after which it translocate across mucosal layers, then colonize in other tissues via the bloodstream [4]. Poultry infections with APEC are frequently associated with sudden death, salpingitis, peritonitis, pericarditis, perihepatitis and airsacculitis. APEC infections also contributed to reduced, quality and hatching of eggs [5,6]. Unfortunately, prophylactic use of antibiotics to control APEC in poultry is still restricted owing to the risk of residues entering the food chain and it’s potential to evolve multi-drug resistant strains [7,8]. Vaccination against APEC can be problematic since, because of differences between disease causing serogroups, many vaccines do not protect well against a heterologous challenge [6]. Furthermore, the strong similarities in genome sequences of APEC strains and human extra-intestinal pathogenic *E*. *coli* indicates that they may also pose a threat to human health and other animals [9].

*Escherichia coli* (*E*. *coli*) is a Gram-negative, rod-shaped, facultative anaerobic bacterium that is commonly found in the lower intestine in endotherms organisms. *E*. *coli* O157:H7 is the most frequently isolated serotype of enterohemorrhagic *E*. *coli* (EHEC) from diseased persons in the United States, Japan, and the United Kingdom [10]. The Centers for Disease Control and Prevention (CDC) has estimated that *E*. *Coli* O157:H7 infections lead to 73,000 sicknesses, 2,200 hospitalizations, and 60 deaths annually in the United States [11]. The annual cost of sickness due to *E*. *coli* O157:H7 infections evaluated by 405 million dollars, including lost productivity, medical care, and premature deaths [12]. This severe effect could be due to that *E*. *coli* O157:H7 is a highly acid-resistant food-borne pathogen that survives in the bovine and human gastrointestinal tracts [13]. Acid resistance is associated with a lowering of the infectious dose of enteric pathogens [14]. The low infectious dose is one of the best known characteristics of *E*. *coli* O157:H7, making this serotypes highly infectious [10].

Gene expression patterns have been examined and described in chickens that differ in its resistance to bacterial infection [15,16], but lack of research has been conducted on the gene expression response to APEC [17,18]. These available studies have been focused on some pathways correlated with pro-inflammatory cytokines, cell death program and immune-competent cells released by/in spleen and peripheral blood leukocytes in broiler and layer chickens. Further, few literatures reported the immune response to lipopolysaccharide on the expression of pro-inflammatory cytokines and leukocytes in the ovary and oviduct of laying and molting hens [19]. However, the knowledge of molecular mechanisms response and pathways of *E*. *coli* related diseases in tissues and organs of commercial layers is relatively scarce. Gene expression of host tissues to infection is commonly utilized to assess and enhance understanding of its response to infection. Information from host tissues and gene expression facilitates the deduction of critical pathways that are important in immune response development.

One of the most important components involved in a wide variety of biological processes is the Mitogen-Activated Protein Kinases (MAPK), serine/threonine-specific protein kinases, that specifically convert extracellular stimuli into a wide range of cellular responses [20]. *P38* MAPK are a class of mitogen-activated protein kinases that are responsive to stress stimuli, such as cytokines, ultraviolet irradiation, heat shock, and osmotic shock, and are involved in cell differentiation, apoptosis and autophagy [21–23]. Disturbances of homeostasis by *E*. *coli* infection activate specific cells like leukocytes, fibroblasts and endothelial cells to release cytokines [24]. Cytokines have been classified into a number of groups based on their activity and the cells they are produced by or act upon, such as interleukins (IL), interferons (IFN), tumor necrosis factors (TNF), transforming growth factors (TGF), migratory inhibitory factors (MIF) and the smaller chemokines. Proinflammatory cytokines, such as interleukin1β (*IL-1β*), play a role in mediating inflammation during disease or injury [25]. *IL-1β* activity was increased in macrophage supernatants from birds suffering from poult enteritis and mortality syndrome [26]. It has been shown that expression of *IL-1β* mRNA was increased 80-fold in the gut of protozoa-infected chickens [27]. The *IL-1β* cytokines act on specific receptors of different target cells leading to a systemic reaction characterized by fever, leukocytosis, and increase in secretion of adrenocorticotrophic hormone (ACTH) and plasma concentration of corticosterone (CORT) [28]. Tumor Necrosis Factor alpha (*TNF-α* or cachetin) is a potent proinflammatory cytokine and is expressed by activated macrophages, lymphocytes, natural killer cells, and epithelial cells [29]. It is also implicated in fever [25] and induces diverse cellular responses that can vary from apoptosis to the expression of genes involved in both early inflammatory and acquired immune responses [30,31]. The release of TNF from chicken macrophages was detected after infection with Marek’s disease virus [32] or protozoa [33]. Injection of chickens with such TNF-like factors increases weight loss, which is partially reversible by treatment with antihuman-TNF antisera [34].

Apoptosis, or programmed cell death, is a multi-pathway biological process that regularly contributes to many physiological and pathological phenomena in multicellular organisms. At the cellular and molecular levels, apoptosis is identified by morphological and biochemical changes such as cell shrinkage, formation of apoptotic bodies, caspase activation, chromatin condensation, and DNA fragmentation [35]. At present, apoptosis regulation is often associated with *caspase-3*, Bcl-2 and *Bax* [36]. The *Bax* gene was the first identified pro-apoptotic member of the Bcl-2 protein family and it promotes apoptosis by binding to and antagonizing the Bcl-2 protein [37]. Many molecular processes of apoptosis are mainly mediated by particular cysteine proteases named caspase, which cleave hundreds of substrates to bring about the typical apoptotic morphology [38]. It has been reported that when caspases, an important mediator of apoptosis [39–41], were inactivated or blocked, the apoptic process was immediately suppressed [42]. In a study by Sun *et al*. [43] to identify genes and pathways that are expressed in bursa of Fabricius of infected chickens with APEC, a strong correlation has been observed between *caspase-3* gene expression and the lesion scores of liver in response to APEC pathology. Therefore, most of treatments to inhibit *Escherichia coli*-induced apoptosis in chickens rely upon the inhibition of *Bax* translocation into mitochondria of infected tissues and prevention of caspase cascade activation [44].

The objective of this research is to gain greater understanding of chicken host response to endotoxin shock induced by the *E*. *coli* infection. mRNA expression of pro-inflammatory cytokines and cell death program genes were examined and analyzed using real-time PCR analysis in both the brain and liver of infected chickens. In addition, the gene toxicity potential of *E*. *coli* was also evaluated by comet assay to determine the induced DNA damage in brain and liver tissues of infected chickens.

## Materials and Methods

### Animals and ethical statement

The current study was conducted in the Poultry Services Center at Faculty of Agriculture, Cairo University. A total number of two hundred and ten of 20-wk-old laying chickens (H&N brown layer hens) were randomly housed in cages, 3 hens per cage, in opened poultry house with photoperiod regimen of 14L: 10D and changed gradually to be 16L: 8D at the experiment time (35 wk of age). A basal diet was formulated according to the recommendations of the National Research Council (NRC, 1994). Water and feed were provided *ad libitum* during the study.

Birds were monitored closely to detect any signs of stress (breathing difficulty, watery discharge of the peak, decreased appetite, ruffled feathers, or droopy looking). The experimental birds were observed every one hour for the first 6 hours, then every 2 hours for the following 6 hours, and every 3 hours for the following 12 hours of the rest of the first day of treatment. For the next 3 days, observation was done every 6 hours. Accordingly, when one or more of these signs appeared, body temperature is measured to determine the action taken. If the body temperature reached 43.5°C or higher, cervical dislocation was used to end the life of these birds. This process was accomplished to minimize suffering of infected birds and to allow humane endpoints. All experimental protocols were approved by Cairo University Ethics Committee for the Care and Use of Experimental Animals in Education and Scientific Research (CU-IACUC).

### Experimental design

Sixty hens were assigned to carry out preliminary sets of experiments to establish the optimal exposure dose of *E*. *coli* and to determine the nearest time of notable response to be used in the remainder studies of this research. Several doses were used in a range of 10^5^−10^9^ colonies/hen, and the body temperature was used as a stress indicator after 2 hours. The highest body temperature, without mortality, was obtained at a concentration of 10^7^ colonies/hen. Hens were injected with this dose and the body temperature was measured after 0, 1, 3, 6, 12, and 24 hours. Increasing in body temperature was detectable as early as 1 hour and was maximal 3 hours after injection. Contingent on the preliminary studies, 10^7^ colonies/hen was used intravenously in a single shut, then tissues and blood samples were collected 3 hours after injection.

At 35 wk of age, a total of 150 hens were randomly assigned into 2 groups with 3 replicates of 25 birds each. Hens of the first group were injected intravenously in the brachial wing vein with 10^7^ colonies/hen *Escherichia coli* O157:H7 in 0.5 ml of sterile saline. The *Escherichia coli* O157:H7 was obtained from the US State Department of Health (Washington D.C.-USA) through Cairo Microbiological Resources Center (Cairo-Egypt). The second group of hens was injected only with 0.5 ml sterile saline and served as a control group. Three hours after injection, the body temperature of 5 hens from each replicate was recorded for both groups; and 10 blood samples were collected from each replicate per group to measure corticosterone concentration in plasma. After that, 5 hens from each group were decapitated and specimens of liver and brain were subjected for RNA extraction protocol. The mRNA expression of protein kinase *p38* gene, pro-inflammatory cytokines genes *IL-1β* and *TNF-α*, and cell death program genes *Bax* and *caspase-3* were analyzed in each of the liver and brain using real time poly chain reaction (RT-PCR) technique. In addition, the DNA damage induced by *E*. *coli* injection in liver and brain cells obtained from 5 hens per group was assessed by a comet assay.

### Stress indicators

Body temperature and plasma corticosterone concentration were used, in both groups, as indicators for stress induced by *E*. *coli* injection. Body rectal temperature was recorded using thermocouple rectal thermometer with a 3-cm insertion probe. Blood samples were withdrawn from the brachial wing vein in heparinized tubes and centrifuged at 2000 x g for 10 min at 4°C. The plasma was separated and stored at -20°C until analyzed. Plasma corticosterone concentration was measured by ELISA reader (BIOTEKELX808) using chicken corticosterone ELISA kits (MyBioSource, San Diego, CA-USA, cat# MBS701668). The intra- and inter-assay coefficient of variations was <8% and <10%, respectively. The analytical sensitivity of the assay was less than 0.0625 ng/ml and the dynamic range of the assay was 0.5–20 ng/ml.

### Total RNA extraction and reverse transcription reaction

Total RNA was extracted from brain and liver tissues using RNeasy Midi Kits (Qiagen, USA) according to manufacturer's instruction. Total RNA was treated with 1 U of RQ1 RNase-free DNase (Invitrogen, Germany) to digest DNA residues, re-suspended in DEPC-treated water. Purity of total RNA was assessed spectrophotometrically at 260/280 nm. The integrity of extracted RNA was determined by using 1.5% agarose gel electrophoresis. Then total RNA was reverse-transcribed into cDNA by using RevertAidTM First Strand cDNA Synthesis Kit (MBI Fermentas, Germany) according to the manufacturer's directions. cDNA was stored at -20°C for relative quantitative real-time PCR.

### Quantitative real-time PCR

PCR reactions were set up in 25 μL reaction mixtures containing 12.5 μL 1× SYBR^®^ Premix Ex Taq^™^ (TaKaRa, Biotech. Co. Ltd., Germany) 0.5 μL 0.2 μM sense primers, 0.5 μL 0.2 μM antisense primer, 6.5 μL distilled water, and 5 μL of cDNA template. The reaction program was allocated to 3 steps of thermal cycling parameters. The first step was set to 95.0°C for 3 min. The second step consisted of 40 cycles in which each cycle divided to 3 steps: (a) at 95.0°C for 15 sec, (b) at 55.0°C for 30 sec, and (c) at 72.0°C for 30 sec. The last step consisted of 71 cycles which started at 60.0°C and then increased by 0.5°C every 10 sec up to 95.0°C. At the end of each qRT-PCR, a melting curve analysis was performed at 95.0°C to check the quality of the used primers. Each experiment included a distilled water control.

The qRT-PCR of *p38*, *IL-1β*, *TNF-α*, *Bax* and *caspase-3* genes were normalized to the main expression of *ß-actin* and transformed using the comparative cycle threshold (CT) method to quantify expression levels as previously described by Ellestad *et al*. [45]. Sequence-specific primers (Table 1) for the real-time PCR were designed using the Primer blast web interface (http://www.ncbi.nlm.nih.gov/tools/primer-blast/index.cgi).

**Table 1:** Details of primers used for real-time PCR quantitative analysis.

Gene symbol	GenBank accession number	Primer sequences
***P38***	CR339030	F:TTGGTTCCACAACTCCAGCACAG
		R:CCGCATCCAGCACCAGCATGT
***IL-1β***	NM_204524	F: GGGCATCAAGGGCTACAA
		R: TGTCCAGGCGGTAGAAGAT
***TNF-α***	BAC55966	F: C ACAGAATGTAAGCCCTGTCC
		R:T GGAGTTCTGCGATCCTGCATT
***Bax***	NM_007527	F:CAGGGTTTCATCCAGGATCGAGCA
		R: TCAGCTTCTTGGTGGACGCATC
***Caspase-3***	GU230786.1	F:TTCAGGCACGGATGCAGATG
		R:TTCCTGGCGTGTTCCTTCAG
***ß-actin***	NM205518	F:TGCGTGACATCAAGGAGAAG
		R:TGCCAGGGTACATTGTGGTA

### DNA damage by comet assay

Brain and liver tissues from chicken were homogenized and isolated by centrifugation (280 g, 15min) in a density gradient of Gradisol L (Aqua Medica, Lodz, Poland). The concentration of the cells was adjusted to (1–3) x 10^5^ cells/ ml by adding RPMI 1640 without glutamine to the single cell suspension. A freshly prepared suspension of cells in 0.75% low melting point agarose (Sigma) dissolved in phosphate buffer saline (PBS; sigma) was cast onto microscope slides pre-coated with 0.5% normal melting agarose and maintained at 37°C. After gelling on a cold metal plate for 1 minute, the cells were then lysed for 1h at 4°C in a buffer consisting of 2.5M NaCl, 100 mMEDTA, 1% Triton X-100, 10mM Tris, and pH10. After the lysis, DNA was allowed to unwind for 40 min in electrophoretic solution consisting of 300mM NaOH, 1mM EDTA, pH>13. Electrophoresis was conducted at 4°C for 30 min at electric field strength 0.73 V/cm (30mA). The slides were then neutralized with 0.4M Tris, pH 7.5, stained with 2ug/ml ethidium bromide (Sigma) and covered with cover slips. The slides were examined at 200 x magnification fluorescence microscope (Nikon Tokyo, Japan) equipped with UV filter block consisting an excitation filter (359nm) and barrier filter (461nm), and connected to a COHU 4910 video camera (Cohu, Inc., San Diego, CA, USA) and a personal computer–based image analysis system (Lucia-Comet v.4.51). Hundred images were randomly selected from each sample (5 hens per group) and the comet tail DNA was measured in brain and liver cells [46]. DNA damages were scored in 4 classes: Class 0 with no tail, Class 1 with tail length < diameter of nucleus, Class 2 with tail length between 1-2X of the diameter of nucleus, and Class 3 with tail length > 2X of the diameter of nucleus [47]. Visual scores of comet tails using the classified classes is presented in (S1 Fig).

### Statistical analysis

All data were represented as mean ± standard deviation of the mean. A Student’s t-test was performed using SPSS 16 (SPSS Inc., Chicago, USA) to calculate the differences between control and *E*. *coli*-treatment groups. A P-value of less than 0.05 was considered significant.

## Results

### Stress indicators

**Fig 1:**
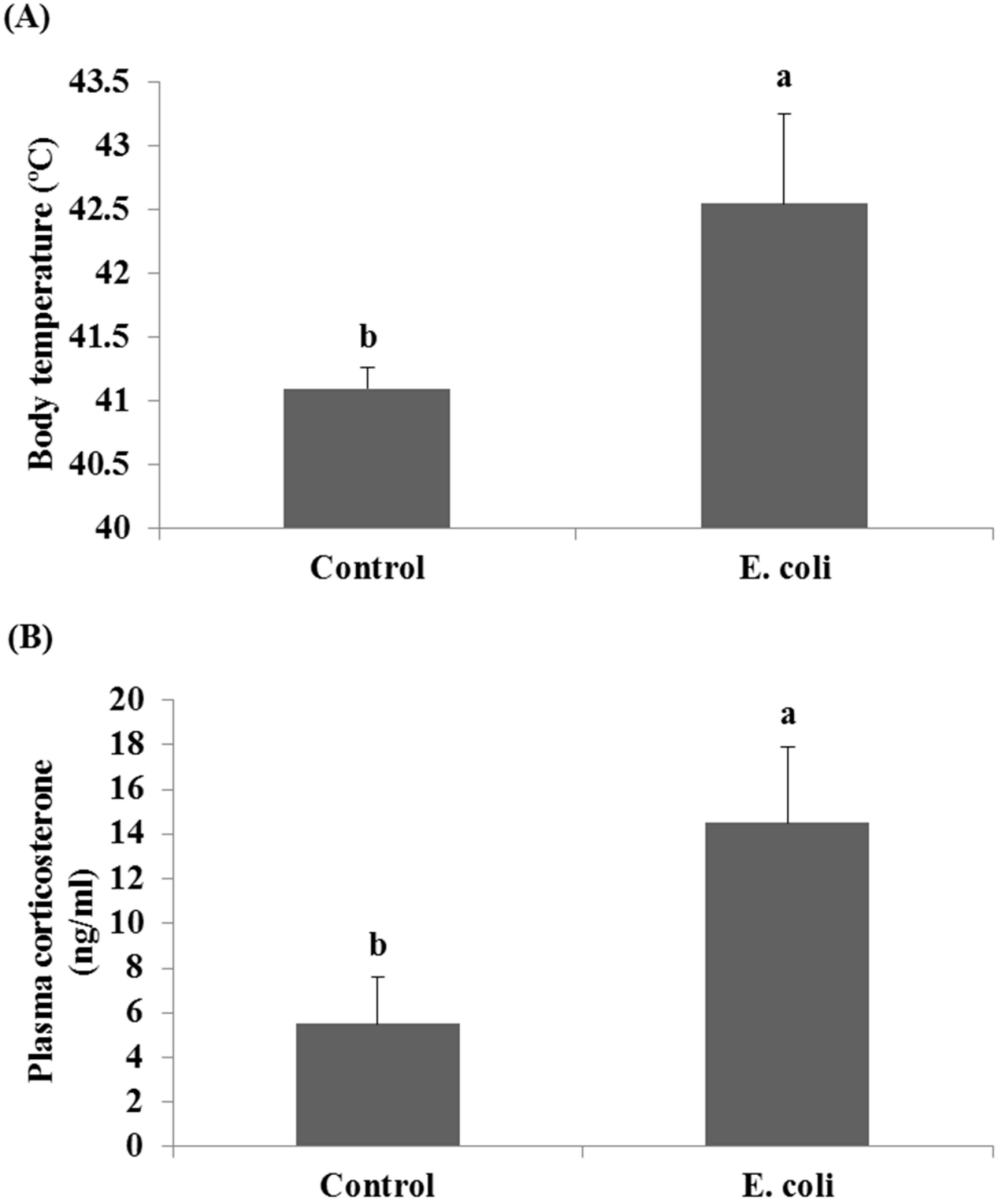
Effect of *E*. *coli* on the body temperature (A) and the plasm corticosterone concentration (B) of chickens. Bars with different letters (a, b) are significantly different at P<0.05.

The effect of *E*. *coli* on the body temperature and the plasma corticosterone concentration as stress indicators of infected chickens has been shown in Fig 1. As shown in Fig 1A, a high significant fever was recorded for infected chickens compared to the control (42.6°C in infected birds *vs*. 41.1°C in control, P<0.05). These infected chickens with *E*. *coli* also expressed a significant (P<0.05) higher concentration in theplasma corticosterone (14.5 ng/ml) than that expressed by the control group (5.5 ng/ml), Fig 1B.

### Quantitative real-time PCR

**Fig 2:**
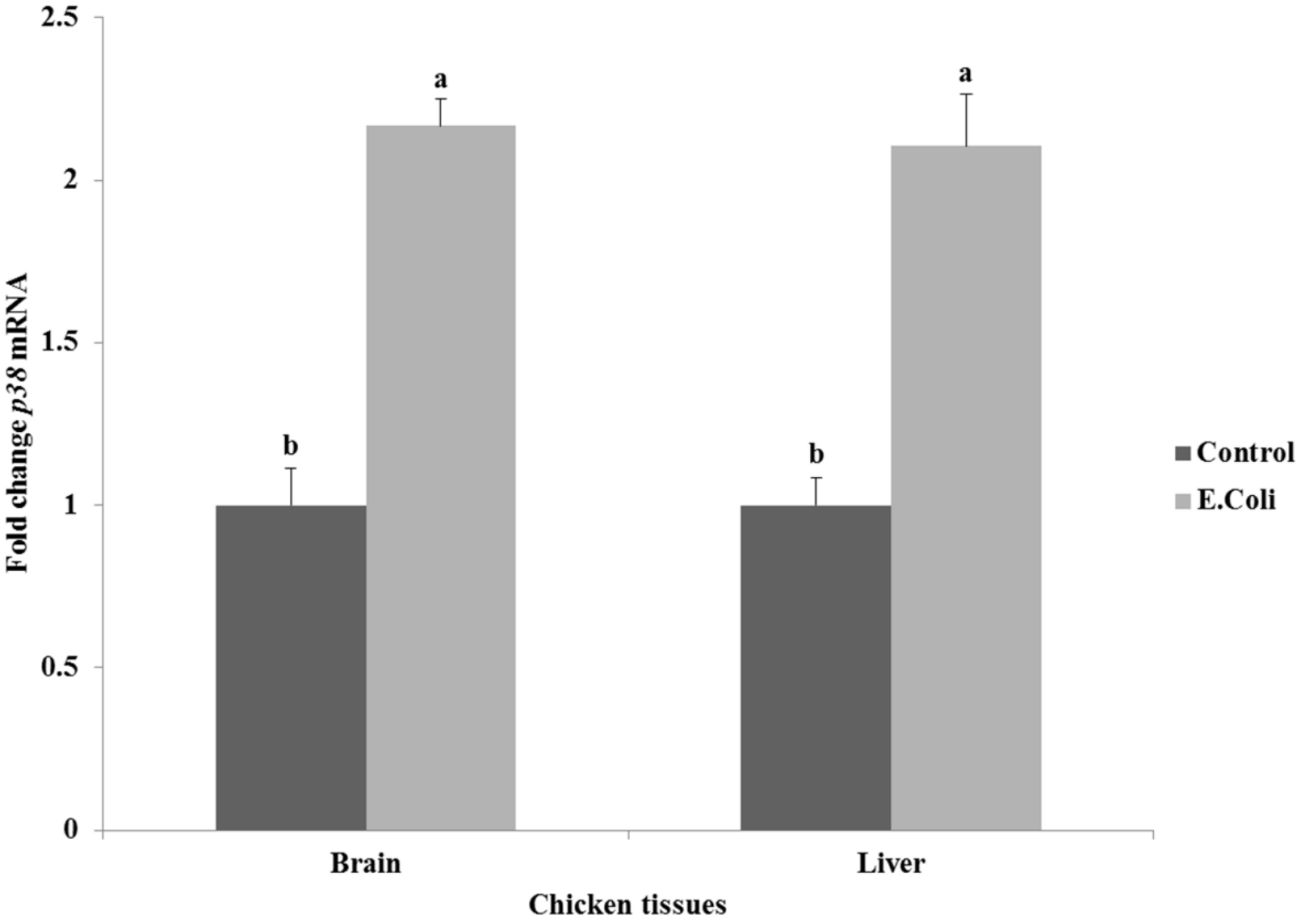
Effect of *E*. *coli* on the relative expression of protein kinase *p38* gene in the brain and liver tissues of chickens. ^a,b^ Mean values within tissue with unlike superscript letters are significantly different (P<0.05).

The effect of *E*. *coli* on the expression of protein kinase *p38* gene in the brain and liver tissues of chickens is summarized in Fig 2. Infection with *E*. *coli* induced significant high expression of *p38* gene by 2.1–2.2 fold (P<0.05) when compared to the control group in both tissues of brain and liver.

**Fig 3:**
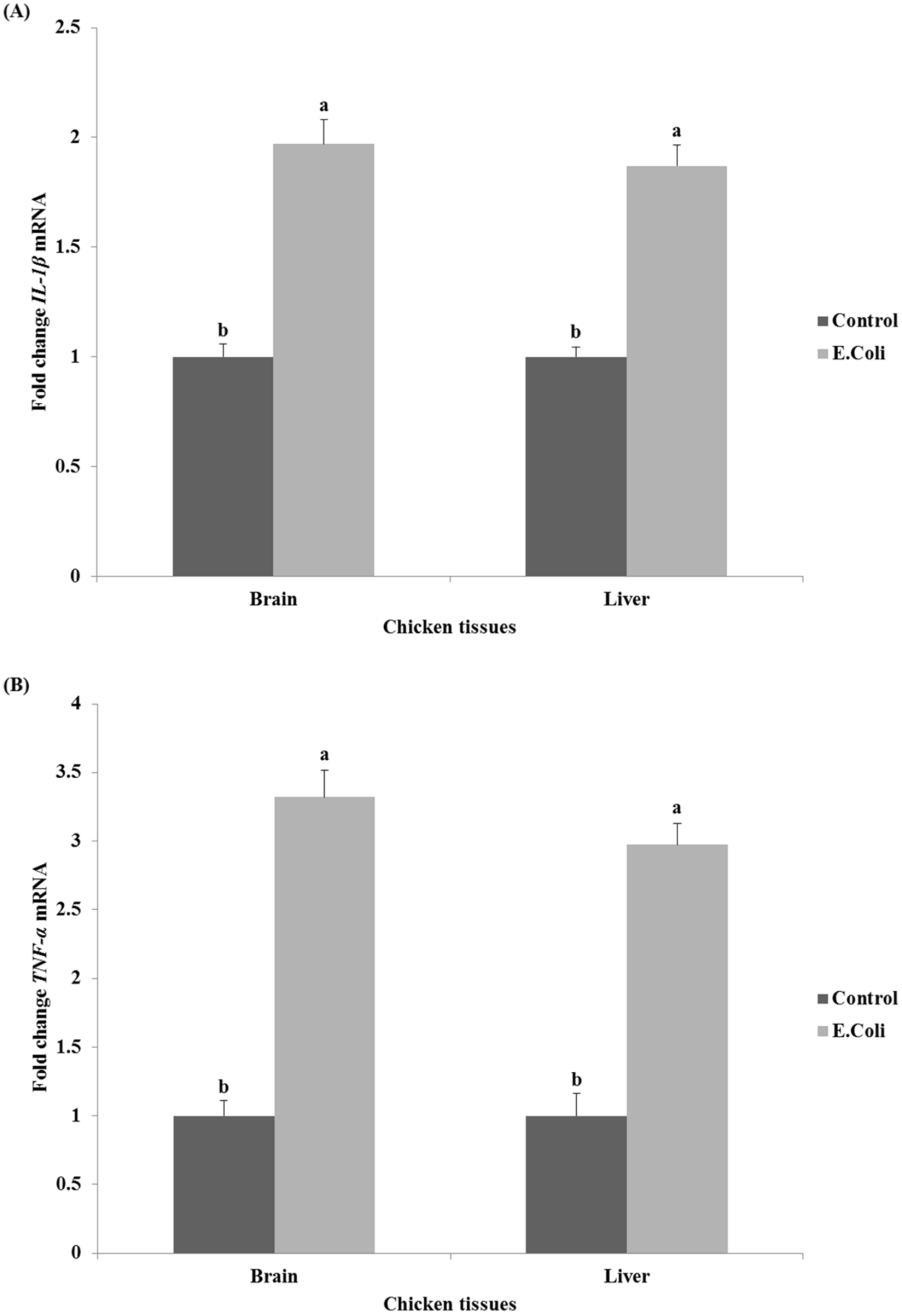
Effect of *E*. *coli* on the relative expression of pro-inflammatory cytokines genes *IL-1β* (A) and *TNF-α* (B) in the brain and liver tissues of chickens. ^a,b^ Mean values within tissue with unlike superscript letters are significantly different (P<0.05).

The relative expression of examined proinflammatory cytokines genes *IL-1β* and *TNF-α* in the brain and liver tissues after *E*. *coli* infection are illustrated in Fig 3. The current data showed that the relative expression of *IL-1β* and *TNF-α* genes followed the same pattern in brain and liver tissues. *IL-1β* gene expression increased significantly (P<0.05) by 2.0 and 1.9 fold in both the brain and liver tissues of infected chickens compared to control chickens, respectively (Fig 3A). Similarly, the expression of *TNF-α* gene increased significantly (P<0.05) by 3.3 and 3.0 fold in the brain and liver tissues of infected chickens compared to control chickens, respectively (Fig 3B).

**Fig 4:**
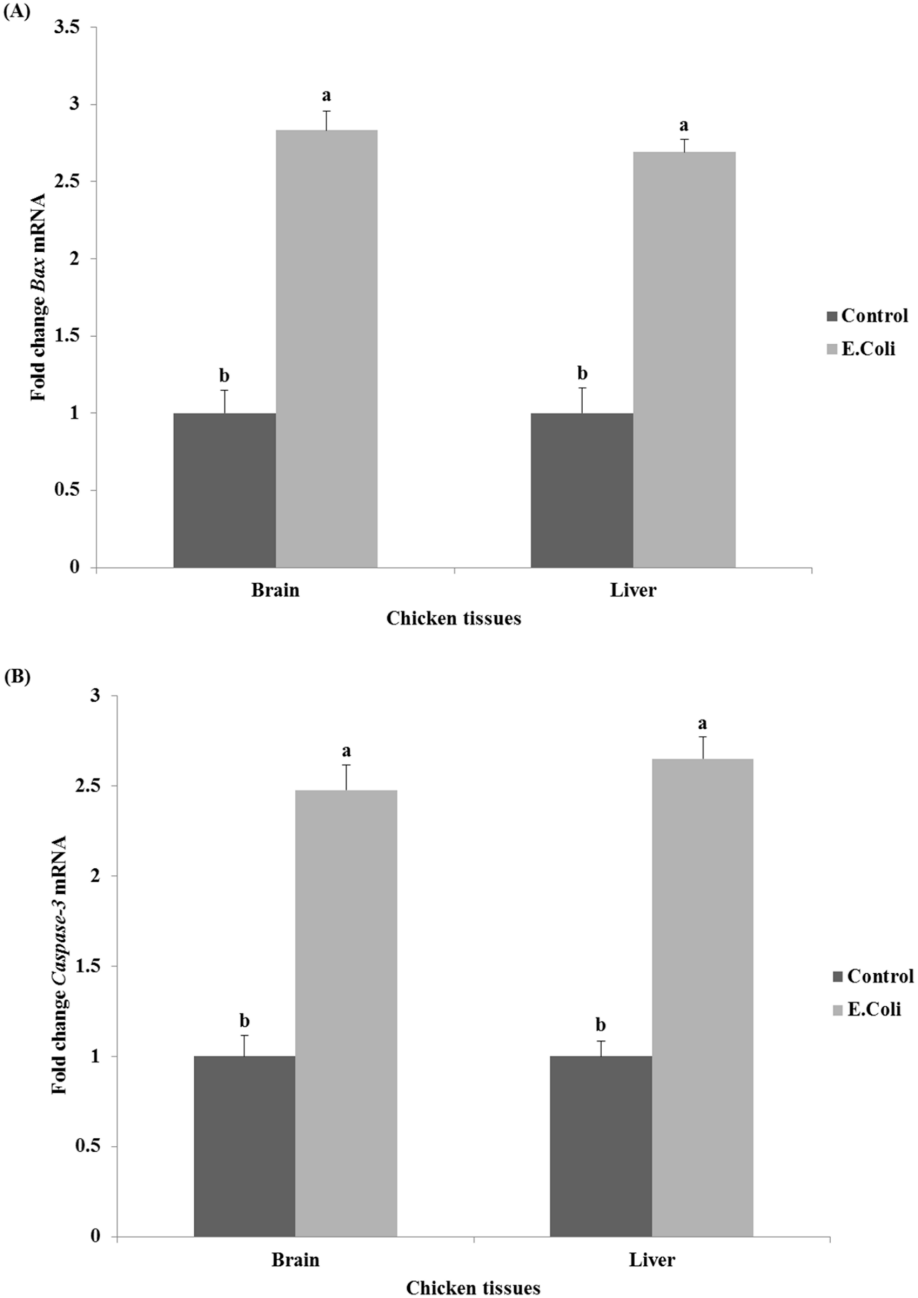
Effect of *E*. *coli* on the relative expression of cell death program genes *Bax* (A) and *caspase-3* (B) in brain and liver tissues of chickens. ^a,b^ Mean values within tissue with unlike superscript letters are significantly different (P<0.05).

The relative expression of examined cell death program genes *Bax* and *caspase-3* in the brain and liver tissues after *E*. *coli* infection are shown in Fig 4. The infected chickens with *E*. *coli* showed a significant (P<0.05) high expression of *Bax* gene in the brain and liver tissues in comparison with their controls (2.7–2.8-fold, Fig 4A). Furthermore, a significant increase in *caspase-3* gene expression was found in the infected chickens (2.5-fold and 2.7-fold in the brain and liver tissues, respectively, P<0.05) when compared to control chickens (Fig 4B).

### DNA Damage detected by Comet assay

Table 2 demonstrates the results of the DNA damages by comet assay in liver and brain tissues of chickens after infection with *E*. *coli*. The chickens treated with *E*. *coli* showed a significant high levels of DNA damage compared with the control chickens (P<0.05). It is also important to note that, the rates of the DNA damage observed in liver tissues (11.2%) were higher than those observed in brain tissues (8.6%) as shown in Table 2. Furthermore, the rate in the infected chicken was generally higher compare to the DNA damage in untreated control chickens that scored in low rates ranged between 4.2 to 4.6 in brain and liver tissues, respectively.

**Table 2:** Visual score of DNA damage in brain and liver tissues of chicken treated with *E*. *coli* using comet assay.

Treatment	No. of animals	No. of cells		Class of comet**				DNA damaged cells (%)
		Analyzed*	Total comets	0	1	2	3	
**Brain**								
**Control**	5	500	21	479	16	5	0	4.2^b^
***E*. *coli***	5	500	43	457	11	14	18	8.6^a^
**Liver**								
**Control**	5	500	23	477	19	4	0	4.6^b^
***E*. *coli***	5	500	56	444	10	22	24	11.2^a^

*No of cells analyzed were 100 per an animal.

**Class 0 = no tail; Class 1 = tail length < diameter of nucleus; Class 2 = tail length between 1X and 2X the diameter of nucleus; and Class 3 = tail length > 2X the diameter of nucleus.

^a,b^ Mean values within tissue with unlike superscript letters are significantly different (P<0.05).

## Discussion

Avian pathogenic *Escherichia coli* (APEC) still exert severe economic loss in poultry industry and remains under focus of researchers and workers in this field. In laying hens, endotoxic shocks and egg production losses caused by *E*. *coli* are still particularly difficult to control yet the nature and consequences of host immune responses to infection are poorly understood, especially in extra-intestinal locations. This research describes the first events of *E*. *coli* interactions with laying chickens host, focusing on the gene expression analysis and the DNA damage which clearly occurred in the brain and liver tissues. To study these events, we carried out preliminary experiments on a group of chickens to determine the best dosage of *E*. *coli* to use in this study and to choose the nearest time of notable response in which chicken expressed high body temperature as a first indicator of stress by infection. We found that injection treatment with 10^7^ colonies of *E*. *coli* per hen is enough to infect the chickens and the first stress indicators were appeared on infected chickens only 3 hours after the treatment.

The results revealed that the body temperature (Fig 1A) and the plasma corticosterone concentration (Fig 1B) were markedly increased in infected chickens when compared with control chickens. Old studies [48–50] reported a febrile response in broiler chickens after administration of lipopolysaccharide (LPS) from *E*. *coli*. We recorded that stress status as high body temperature in infected chickens begins following the 3 hr of *E*. *coli* injection. The stress status was associated with a significant increase in plasma corticosterone concentration. The corticosterone is the end product of the neuroendocrine molecules secreted from hypothalamic-pituitary-adrenal axis as an integrated response to stress [51]. These hormones can therefore modulate the activities of immune cells; in particular the production of proinflammatory cytokines and chemokines, which themselves can in turn modulate the activity of the hypothalamus and thus alter hormone production [52]. Besides the role of corticosterone in enhancing the formation of antibodies and the humoral-mediated immune response [53,54], it also mobilizes and produces glucose to meet the increased energy requirement during stress [55].

As shown in Fig 2, the infection with *E*. *coli* induced significant (P<0.05) high relative expression of *p38* gene when compared with control in both tissues of brain (2.2-fold) and liver (2.1-fold). Our results are consistent with previous studies by Dziarski *et al*. [56] who reported that LPS produced by bacteria strongly activates all kinases, and Cao *et al*. [23] who reported also an increase in MAPK1 expression as a result of bacterial infection in ducks. Mitogen-activated protein kinases are involved in directing cellular responses to a diverse array of stimuli such as mitogens, osmotic stress, heat shock, and pro-inflammatory cytokines [20], and the *E*. *coli* infection in our study. They regulate gene expression, mitosis, proliferation, differentiation, cell survival, and apoptosis [22].

The significant increase in *p38* gene expression in brain and liver of infected chickens was accompanied with a significant increase in the pro-inflammatory cytokines genes expression studied in our experiment. This elevated expression of pro-inflammatory cytokines may be the prerequisite to prevent the development of infection [19]. The current data showed that the relative expression of *IL-1β* in both the brain and liver tissues were significantly increased (P<0.05) approximately by 2-fold in infected chickens when compared to control chickens (Fig 3A). These results are in line with previous studies [26,27,57,58] where the *IL-1β* activity and mRNA expression has been shown to increase after viral and bacterial infections in the chicken. Moreover, Shaughnessy *et al*. [59] reported an increase in the expressions of *IL-1β* in avian intestinal tissues in response to *Salmonella* and *Campylobacter* bacteria. The study of Nii *et al*. [19] demonstrated that the *IL-1β*expression was significantly up-regulated in both uterus and vagina of White Leghorn laying hens at 3 hr after LPS injection. Munyaka *et al*. [60] saw a similar response in the spleen and cecal tonsils of laying hens. At the same time, the relative expression of *TNF-α* gene significantly (P<0.05) increased by approximately 3.0–3.3-fold in the brain and liver of infected chickens when compared with their controls (Fig 3B). *TNF-α* (also known as cachetin) is a primary regulator of both the immune response and inflammation [61]. As previously observed by Tallant *et al*. [62] and Guma *et al*. [63], the increase in *TNF-α* gene expression as a result of *E*. *coli* infection may itself be a reason for the increase in *p38* gene expression in the same group (Fig 2); and these two genes are essential to trigger the inflammation in infected chickens.

In the present study, we found that the relative expression of *Bax* gene was significantly increased (P<0.05) by 2.8-fold in both the brain and liver tissues of infected chickens when compared with untreated control chickens (Fig 4A). Some research and studies were found about *Bax* expression in chickens infected by *E*. *coli*, as it reported by Gao *et al*. [44] who found that *Clostridium butyricum* possesses the ability to prevent *Escherichia coli*-induced apoptosis in chicken embryo intestinal cells; and Sandford *et al*. [17] who reported changes in apoptosis-related genes in the challenged-susceptible birds. These reports supported our results concerning the main role of *Bax* in apoptosis after *E*. *coli* infection. We also observed a significant increase by 2-5-2.7-fold in *caspase-3* gene expression in the tissues of infected chickens (P<0.05; Fig 4B). Similar results were obtained by Bastiani *et al*. [64] who found a strong activity of *caspase-3* in a murine macrophages cell line 2 hr after incubation with an APEC strain. Recently, Sun *et al*. [43] revealed that novel pathways for apoptosis in bursa of Fabricius of susceptible chicken in response to APEC infection leads to the activation of *caspase-3* and ends at the release of pro-apoptotic protein *Bax*.

On the other hand, the rates of DNA damage observed by comet assay in liver and brain tissues (11.2% and 8.6%, respectively) were higher in chickens treated with *E*. *coli* than those observed in untreated control chickens (ranged between 4.2 to 4.6 in brain and liver tissues, respectively; Table 2). Our results are in agreement with previous findings [65] which demonstrated that infection of eukaryotic cells with *E*. *coli* strains induced host-cell DNA double-strand breaks and activation of the DNA damage signaling cascade. The apoptotic morphology (plasma membrane blebbing, cytoplasm vacuolization, chromatin condensation and DNA degradation) is essentially the result of the proteolytic action of caspases family upon specific cellular substrates [66,67]. Such action of caspases could be also seen in our study hence the *caspase-3* expression and DNA damage were significantly (P<0.05) high in infected chickens (Fig 4B and Table 2). Moreover, *Bax* expression was significantly increased in the same group of infected chickens (Fig 4A). It is thought that *Bax* is still required to activate effector caspases, particularly *caspase-3/-9* activation, to accomplish DNA damage and apoptosis [68]. Other scientists explored that the *Bax* gene in primary neuronal cultures derived from mouse cortex was highly expressed after the DNA damage by overexpression of other mediator genes such as Peg3/Pw1and P53 (but not caspase pathways), resulting in neuronal cell death [69]. Such hypothesis was previously evidenced in neuronal cells of the ovary, placenta, testis and brain in mouse and human [70–72], and could explain the high expression of *Bax* gene accompanied with DNA damage incidence in the brain of infected chicken with *E*. *coli* in our study. *TNF-α* may also play a role in the DNA damage observed in our study. Rahman and McFadden [29] explained that TNF ligand homotrimer binds to the extracellular receptors to initiate some intracellular signaling pathways; including caspase family activation, which leads to cell death [73,74].

Referring back to the fever which occurred in the infected chickens in this study (Fig 1A), we think that this fever has been attributed to the endogenous pyrogenic action induced by the high *IL-1β* expressed in these chickens [25]. It may be also due to the high expression of *TNF-α* in infected chickens which has been implicated as a key mediator of fever in several animal models [25]. Pro-inflammatory cytokine genes including *IL-1β* and *TNF-α* were also studied in many organs of chicken embryos infected by mycoplasma disease [75]. They found that these genes were significantly up-regulated in the liver and spleen of infected embryos by macrophages and cells of the reticuloendothelial system; therefore, we also see from our results that *E*. *coli* infection in laying chickens may cause proliferation of similar repertoire of immune cells in the reticuloendothelial system of liver and brain tissues. Similar events were previously concluded in a work by Horn *et al*. [76] that endothelial and epithelial cells, heterophils, and macrophages, which were localized in lung tissues of infected chickens with avian *E*. *coli*, were involved in the defense and died at the infection sites. If this also happens in our experimental model, we can understand the activation of cell death program in liver and brain tissues after chickens’ infection with *E*. *coli*, wherein the *Bax* and *caspase-3* genes were highly expressed (Fig 4). The proliferated macrophages in liver and brain tissues of infected chickens may participate in the uptake and digestion of *E*. *coli* bacteria as one of the known macrophages’ functions [77,78]. It was strongly suggested that upon uptake and digestion of *E*. *coli* bacteria, the *caspase-9*- and *caspase-3*-dependent branch of the apoptotic pathway was activated in a murine macrophages cell line [79]. Finally, the proteolytic action of caspases family on specific cell substrates [66,67] led to the apoptotic morphology aspects including DNA damages we detected in the infected chickens at the present study (Table 2).

In conclusion, the current study provides more understanding to APEC infection in chickens at cellular and molecular levels. The proinflammatory cytokine genes *IL-1β* and *TNF-α* are highly expressed accompanied with an increase in MAPK-*p38* gene expression in the brain and liver tissues of infected chickens. Moreover, high expression of *Bax* and *caspase-3* genes has been occurred in infected chickens associated with programmed cell death and DNA damage in brain and liver tissues. Further studies are required to clarify if such responses are destructive or protective, and such information may set the means through which a chicken is able to mount a successful defense against APEC.

## Supporting Information

S1 Fig Visual score of DNA damage using comet assay in chicken treated with *E*. *coli*. Class scores (0–3): Class (0) = no tail, Class (1) = tail length < diameter of nucleus, Class (2) = tail length between 1X and 2X the diameter of nucleus, and Class (3) = tail length > 2X the diameter of nucleus. (Original magnification: 200x; Scale bars: 50 μm).
(TIF)
