# Supplementary figures and images for: Expression of Inflammatory and Cell Death Program Genes and Comet DNA Damage Assay Induced by Escherichia coli in Layer Hens
Source: PLoS One. 2016 Jun 27;11(6):e0158314. doi: 10.1371/journal.pone.0158314 (PMC4922552; doi:10.1371/journal.pone.0158314)

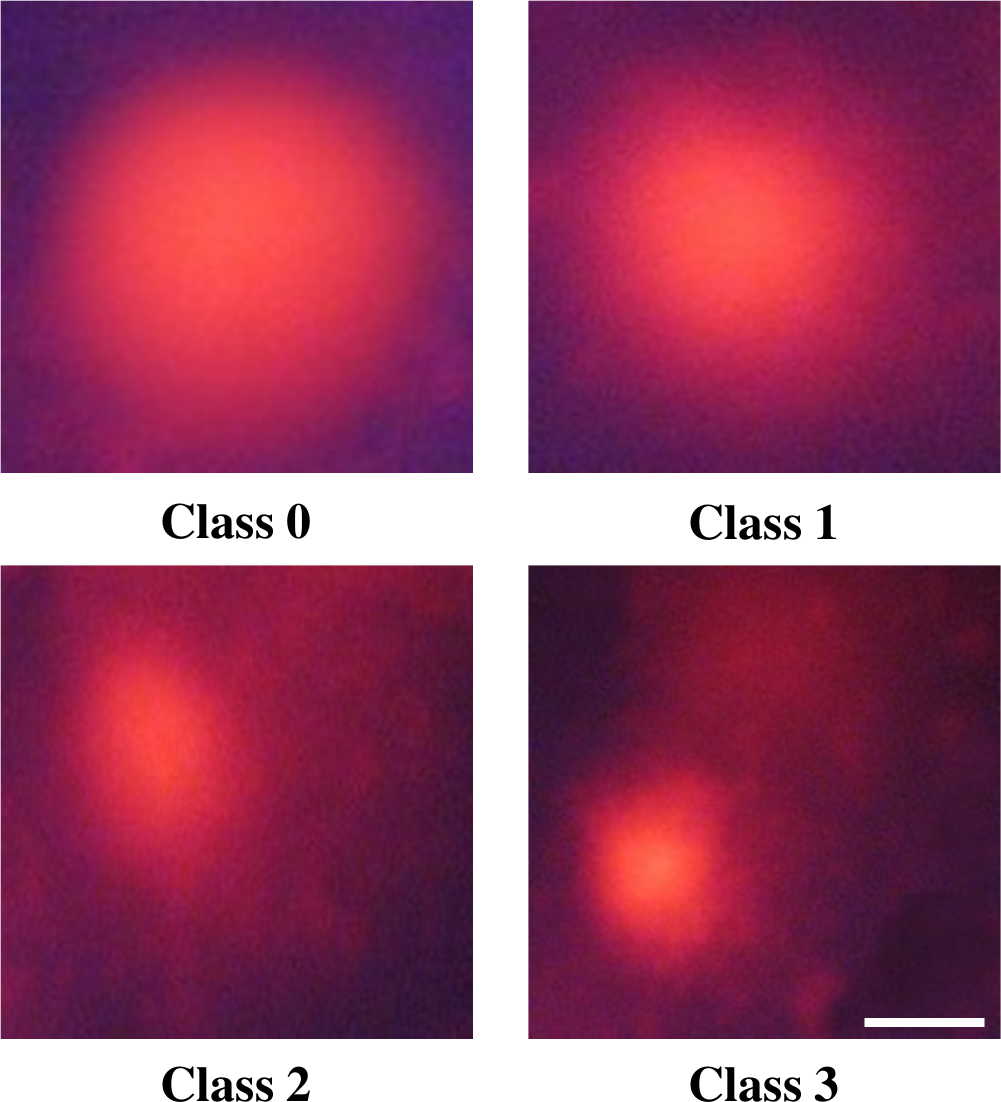

Supplement: S1 Fig — Class scores (0–3): Class (0) = no tail, Class (1) = tail length < diameter of nucleus, Class (2) = tail length between 1X and 2X the diameter of nucleus, and Class (3) = tail length > 2X the diameter of nucleus. (Original magnification: 200x; Scale bars: 50 μm). (TIF) [file pone.0158314.s001.tif]
